# New trends for overcoming ABCG2/BCRP-mediated resistance to cancer therapies

**DOI:** 10.1186/s13046-015-0275-x

**Published:** 2015-12-30

**Authors:** David Westover, Fengzhi Li

**Affiliations:** Department of Pharmacology and Therapeutics, Roswell Park Cancer Institute, Elm and Carlton Streets, Buffalo, NY 14263 USA

**Keywords:** ABCG2, resistance, Efflux, Chemotherapy, Cytotoxic, Targeted agent, PDT, Radiation

## Abstract

ATP-binding cassette (ABC) transporters make up a superfamily of transmembrane proteins that play a critical role in the development of drug resistance. This phenomenon is especially important in oncology, where superfamily member ABCG2 (also called BCRP – breast cancer resistance protein) is known to interact with dozens of anti-cancer agents that are ABCG2 substrates. In addition to the well-studied and well-reviewed list of cytotoxic and targeted agents that are substrates for the ABCG2 transporter, a growing body of work links ABCG2 to multiple photodynamic therapy (PDT) agents, and there is a limited body of evidence suggesting that ABCG2 may also play a role in resistance to radiation therapy. In addition, the focus of ABC transporter research in regards to therapeutic development has begun to shift in the past few years. The shift has been away from using pump inhibitors for reversing resistance, toward the development of therapeutic agents that are poor substrates for these efflux pump proteins. This approach may result in the development of drug regimens that circumvent ABC transporter-mediated resistance entirely. Here, it is our intention to review: 1) recent discoveries that further characterize the role of ABCG2 in oncology, and 2) advances in reversing and circumventing ABC transporter-mediated resistance to anti-cancer therapies.

## Background

Resistance to anti-cancer therapies is one of the most studied subjects in modern biomedical research [1, 2]. Though many mechanisms of drug resistance have been identified in cancer, drug efflux mediated by xenobiotic transporters is one of the best validated. ATP-binding cassette (ABC), sub-family G, isoform 2 protein (ABCG2, also known as breast cancer resistance protein, BCRP) is a drug efflux pump and an important member of the ABC transporter superfamily. ABCG2 was identified independently by three separate groups in 1998 and 1999 [3–5]. In normal tissue, ABCG2 performs a multitude of functions. ABCG2 is expressed at very high levels in the placenta and protects the developing fetus from endo- and exotoxins [5]. ABCG2 is also found at the blood-brain barrier, where it likewise protects the brain from harmful compounds [6]. ABCG2 also regulates the homeostasis of nutrients and certain hormones. In the gastrointestinal tract, ABCG2 plays a role in nutrient absorption [7]. ABCG2 helps to concentrate vitamins and possibly hormones in breast milk [8], and may regulate testosterone levels in the prostate, as ABCG2 is expressed in normal prostate basal epithelial cells [9]. The sebaceous glands, exocrine glands located in the skin that secrete sebum to lubricate and waterproof skin and hair, also express very high levels of ABCG2 [10].

### ABCG2 and cancer

Two of the three groups that initially isolated ABCG2 did so while investigating resistance to anti-cancer agents that had developed in cell culture [3, 4]. Since ABCG2 was first identified in drug-resistant cancer cells, it was hypothesized that a variety of cytotoxic agents were substrates for ABCG2, and that resistance to these agents was the result of drug efflux by ABCG2 [3, 4]. Though not the focus of this review, we have summarized a portion of the literature in regards to ABCG2 and cytotoxic or targeted therapies (Table 1) [11–16]. Several excellent reviews cover these findings in greater detail [17–20].

**Table 1:** Brief summary of anti-cancer compounds that are ABCG2 substrates

Compound	Mechanism of action	Note (see below)	Reference
Traditional cytotoxics			
Mitoxantrone	Topoisomerase II poison		[4]
Etoposide	Topoisomerase II poison		[11, 12]
Doxorubicin	DNA intercalator; Topo II poison	1	[27, 29]
Daunarubicin	DNA intercalator; Topo II poison	1	[27, 29]
Epirubicin	DNA intercalator; Topo II poison	1	[29]
Topotecan	Topoisomerase I poison		[22]
Irinotecan	Topoisomerase I poison		[27]
SN-38	Topoisomerase I poison	2	[21]
5-fluorouracil	Thymidylate synthase inhibitor		[26]
Methotrexate	Dihydrofolate reductase inhibitor		[24]
Cladribine	Nucleoside analogue		[28]
Clofarabine	Nucleoside analogue		[28]
6-mercaptopurine	Nucleoside analogue		[28]
Flavopiridol	CDK9 inhibitor		[25]
Tyrosine kinase inhibitors			
Imatinib	Bcr-Abl inhibitor		[13, 89]
Dasatinib	Bcr-Abl inhibitor	3	[14, 40]
Nilotinib	Bcr-Abl inhibitor		[14]
Sorafenib	Multi-kinase inhibitor	4	[91, 93]
Sunitinib	Multi-kinase inhibitor	5	[87, 92]
Gefitinib	EGFR inhibitor		[15]
Erlotinib	EGFR inhibitor		[16]
Rucaparib	PARP inhibitor	6	[37, 38]
PDT agents			
Pheophorbide a	Photosensitizer		[39]
Chlorin e6	Photosensitizer		[41]
HPPH	Photosensitizer		[42, 43]
5-aminolevulinic acid	Photosensitizer		[48]
Porfimer sodium	Photosensitizer	7	[49]

1, Requires gain-of-function mutation at ABCG2 R482

2, SN-38 is the active metabolite of irinotecan

3, One study suggests ABCG2 expression correlates with poor patient response

4, Phase I/II trial of sorafenib plus irinotecan recently completed

5, Phase III trial of sunitinib plus FOLFIRI found no benefit over FOLFIRI alone

6, ABCG2 also limits rucaparib oral bioavailability

7, ABCG2 correlates with poor response to porfimer sodium in NSCLC patients

Multiple in vitro studies have demonstrated that methotrexate, mitoxantrone, flavopiridol, 5-fluorouracil, as well as the camptothecin analogues topotecan, irinotecan, and SN-38 are all substrates for ABCG2, and high expression of ABCG2 correlates with decreased intracellular accumulation of these compounds and consequentially a decrease in drug potency [4, 21–27]. A number of nucleoside analogues in clinical use are also known to interact with ABCG2 [28]. In addition, gain-of-function mutations in *ABCG2* (R482G and R482T) result in efflux of anthracyclines [29, 30]. A number of clinical studies have also observed correlations between high ABCG2 activity and decreased survival [31–33], and links between failure of a variety of cytotoxic and targeted therapies with ABCG2 activity [34–36]. Most recently, for example, ABCG2 has been shown to transport rucaparib [37], a PARP inhibitor under clinical investigation, as well as limit its oral bioavailability [38].

### ABCG2 and photodynamic therapy

Photodynamic therapy (PDT) uses tumor-selective photosensitizers and subsequent activation by light of a specific wavelength to generate reactive oxygen species. These species, in turn, damage cancer cells and induce apoptosis and necrosis. Currently, PDT agents are approved in the U.S. for the treatment of esophageal cancer and non-small cell lung cancer. A number of PDT agents are known to be substrates of ABCG2. High ABCG2 expression decreases the intracellular accumulation and in vitro potency of the investigational PDT agents pheophorbide a [39, 40], pyropheophorbide a methyl ester [41], and chlorin e6 [41]. There is also in vitro and *in vivo* evidence that ABCG2 can cause resistance to a PDT agent currently under clinical investigation, 2-(1-hexyloxethyl)-2-devinyl pyropheophorbide, commonly known as HPPH [42, 43]. There is also evidence that clinically used PDT agents are substrates for ABCG2. Interest in 5-aminolevulinic acid (ALA) has been growing in recent years, as ALA is now used as a fluorescent aid during tumor resection in glioma patients [44] as well as a photosensitizer for PDT of pre-cancerous actinic keratosis [45, 46]. Robey et al. initially identified ALA as a substrate for ABCG2, and recent work by two other groups has further confirmed their findings [41, 47, 48]. Lastly, Usuda et al. reported that the potency of porfimer sodium is decreased in response to high ABCG2 activity [49]. Porfimer sodium is a photosensitizer that is approved by the FDA for the treatment of esophageal cancer and endobronchial non-small cell lung cancer lesions. Additionally, Usuda and colleagues reported that lung cancer patients with localized disease who expressed high levels of ABCG2 protein responded worse to porfimer sodium than patients with lower levels of ABCG2 [49], which further validated ABCG2 as a clinically-relevant mechanism of resistance in cancer.

### ABCG2 and cancer stem cells

ABCG2 has also been implicated in another realm of cancer that is somewhat separate from treatment response: the cancer stem cell (CSC) phenotype. CSCs are a subset of cancer cells that share properties with “normal” stem cells: self-renewal and the ability to differentiate into multiple types of cells (reviewed in [50]). CSCs have been hypothesized to play a role in tumorigenesis, resistance, recurrence, metastasis, and tumor heterogeneity [50–52]. CSCs are often isolated or identified by detection of cell surface markers, including CD44, CD24, CD133, and others [50, 53], and also by detection of aldehyde dehydrogenase activity [54]. However, another method of isolating CSCs has been through the identification of a subpopulation that is able to efflux chemotherapeutics or, more commonly, the dye Hoechst 33342, an ABCG2 substrate [55].

The Hoechst 33342 efflux assay was developed by Dr. Margaret Goodell and colleagues who were attempting to use the DNA binding dye to measure DNA content in cycling bone marrow cells [55]. After treating murine bone marrow cells with Hoechst 33342, the group excited the cells with an ultraviolet laser and recorded emission at two wavelengths using a 450/20 nm band pass filter (the standard filter for evaluating DNA content using Hoechst 33342) and a 675 nm long pass edge filter [55]. Simultaneously displaying emission at both wavelengths allowed the team to identify a population of cells that was removed from the main body which they hypothesized was the result of dye efflux mediated by molecular efflux pumps [55]. This “side population”, as they called it, contained cells that were enriched for hematopoietic stem cell markers and were better able to repopulate the bone marrow of mice after radiation [55]. Several groups were able to later identify ABCG2 as a key contributor to Hoechst 33342 efflux and an important side population marker [56–59]. Since then, the Hoechst 33342 efflux assay has been used to successfully isolate a number of normal stem-like populations, such as retinal stem cells [60] and primitive neural cells [61], as well as CSC-like populations in lung cancer cell lines [62], head and neck cancer cell lines [63], hepatocellular carcinoma cell lines [64], a glioma cell line [65], primary neuroblastoma cultures [66], ovarian cancer cell lines [67], and in ascites cells from patients with ovarian cancer [67].

ABCG2 is often associated with CSCs because of the presence of ABCG2-positive cells in the side population identified by dye efflux assays. However, several groups have concluded that ABCG2 is not a defining feature of all stem-like populations. For example, *ABCG2* deficiency does not prevent normal hematopoietic development in mice [56], and CSC populations isolated using cell surface markers do not always express ABCG2 [68]. Additionally, ABCG2-negative cancer cells are able to form tumors in breast, prostate, colon, and glioma xenograft models at the same rate as ABCG2-positive cancer cells [69]. However, a number of reports have identified two areas of interest where ABCG2 may be functionally relevant in regards to CSCs and therapeutic response: in castration-resistant prostate cancer, and in radiation resistant cancer cells.

### ABCG2 and hormone-refractory cancers

Some groups have hypothesized that, although not all CSCs are ABCG2-positive, ABCG2-positive cancer cells with at least some stem-like qualities become more relevant during the development of treatment resistance. In addition to resistance to treatment modalities discussed earlier, this would also allow for resistance to hormone-based therapies, as ABCG2 has been shown to efflux a number of androgenic and estrogenic hormone conjugates [70–73]. This phenomenon has been studied most extensively in the prostate cancer, where ABCG2 is highly expressed in a small population of stem-like cancer cells and is able to regulate the efflux of androgen [72]. Overexpression of ABCG2 has also been shown to promote a stem-like phenotype in prostate cells [74]. These cells are believed to be less responsive to androgen, as they do not express detectable levels of the androgen receptor. Further, Gu et al. demonstrated that androgen receptor-negative prostate CSCs can repopulate tumors in animals following serial implantation [75]. Based on this evidence, it has been hypothesized that this population of androgen-independent cells may be the progenitor cells that are responsible for the development of castration-resistance disease: as androgen deprivation therapy induces apoptosis in androgen-dependent cells (reviewed in [76]), the tumor could potentially be repopulated by CSCs that are not reliant on androgen.

### ABCG2 and radiation therapy

Studies have noted that a number of ABC transporters are often upregulated in cells that are resistant to radiation therapy [77–79]. Conventional wisdom was that these pumps were not actively radioprotective, but were instead indicative of the stem-like phenotype common in radiation resistance populations of cells. However, at least one group had proposed a functional role for ABC transporters in radiation resistance as early as 2007, positing that efflux pump-mediated glutathione modulation could play a role in radiation resistance [80]. Though that specific hypothesis has remained largely unstudied, a small number of reports have emerged that may suggest a functional role for ABC transporters, including ABCG2, in radiation resistance. In 2009, Ning et al. published a report detailing a population of stem-like cells in the bladder cancer line T24. They reported that this stem-like population, isolated by Hoechst 33342 efflux and expressing higher *ABCG2* mRNA than unsorted T24 cells, was resistant to radiation treatment [81]. Intriguingly, administration of verapamil, an inhibitor of ABC transporters as well as an inhibitor of calcium and potassium transporters, sensitized these cells to radiation treatment [81]. More recently, Ingram and colleagues reported high ABCG2 expression in medulloblastoma cells that survived exposure to long-term low-dose radiation as well as short-term high-dose radiation [82]. They observed a reversal of radioresistance following treatment with R-verapamil, which has weaker anti-ion channel activity compared to S-verapamil, yet retains its ability to inhibit ABC transporters. Curiously, they did not see reversal of radioresistance following treatment with specific inhibitors of ABCG2, such as Ko143 [82]. The authors interpreted this as evidence for redundancy of ABC transporters in regards to transporter-mediated radiation resistance, though they did not rule out residual anti-ion channel activity [82]. In our view, more studies are needed before a functional role for ABC transporters in radioresistance can be conclusively established.

### Therapeutic strategies to overcome ABCG2-mediated resistance

#### Inhibition of ABCG2 activity

Since the discovery of ABCG2, researchers have been investigating methods to reverse or circumvent ABCG2-induced resistance. One attractive possibility was to inhibit ABCG2 activity, thereby halting drug efflux. This strategy initially showed promise in vitro where potent inhibitors of ABCG2, such as fumitremorgin C, were able to restore the potency of ABCG2 substrates [83, 84]. However, attempts to translate this strategy to the clinic have been unsuccessful so far. The first problem to arise was the toxicity of first-generation ABCG2 inhibitors in animal models. Fumitremorgin C, for example, causes severe neurotoxicity [85]. Other groups have reported that less toxic inhibitors of ABCG2, such as tariquidar, may not result in clinically beneficial increases in drug accumulation [86]. Interestingly, results from studies using tyrosine kinase inhibitors that also inhibit ABCG2 activity are more encouraging.

Many tyrosine kinase inhibitors that are substrates for ABCG2 are also able to function as competitive inhibitors of ABCG2-mediated efflux of cytotoxics, as the two drug classes share a common binding site [87, 88]. Like fumitremorgin C, several tyrosine kinase inhibitors, including sunitinib, nilotinib, sorafenib, and imatinib, are able to sensitize cells with high ABCG2 expression to cytotoxics which are ABCG2 substrates, including topotecan and SN-38 [89, 90]. Mazard *et al*. were able to demonstrate that sorafenib enhanced the intracellular accumulation of SN-38 and that combination treatment of sorafenib with irinotecan improved survival of mice implanted with irinotecan-resistant tumors [91]. However, clinical trials using tyrosine kinase inhibitors as ABCG2 inhibitors or in combination with cytotoxic agents that are ABCG2 substrates have shown mixed outcomes. A phase III trial of sunitinib in combination with the FOLFIRI regimen was discontinued due to increased adverse events, including increased drug toxicity-related deaths, in the sunitinib-treated arm with no benefit to overall survival [92]. However, a phase I/II trial of sorafenib in combination with irinotecan in patients who had previously failed irinotecan-containing regimens was concluded in 2014 with promising results [93].

#### Inhibition of ABCG2 expression as an emerging therapeutic option

Another proposed strategy in overcoming ABCG2-mediated resistance to chemotherapeutics was to inhibit ABCG2 protein expression. The concept of inhibiting ABC transporter expression is not new; Hiroyuki Kobayashi and colleagues first reported on hammerhead ribozymes that were able to target *ABCG2* mRNA in 1994 [94]. More recently, RNA interference (RNAi) has been used to knock down ABCG2 expression in cell culture and restore therapeutic benefit to anti-cancer agents that are ABCG2 substrates [95–98]. While there was some doubt for a number of years about the practicality of RNAi translating to clinical use [99, 100], the reports in the last five years of successful cleavage of targeted sequences in humans using nanoparticle delivery of siRNA particles [101, 102] opens the door for renewed interest in the potential application of RNAi as a means of disrupting multi-drug resistance mediated by ABC transporters.

Alternatively, several groups have proposed pharmacologic inhibition of ABCG2 expression as a potential tool to combat drug resistance. Again, tyrosine kinase inhibitors have been used to demonstrate proof of principle in vitro. Nakanishi *et al*. demonstrated that tyrosine kinase inhibitors that target the PI3K-Akt pathway, such as LY294002, are able to downregulate ABCG2 expression, thereby sensitizing cells to therapeutic agents that are ABCG2 substrates [103]. Other groups have investigated the use of phosphodiesterase-5 inhibitors for the same purpose. One phosphodiesterase-5 inhibitor, sildenafil, has shown promise as an inhibitor of multiple ABC transporters at clinically achievable levels [104, 105]. One report describes the reversal of methotrexate, mitoxantrone, and paclitaxel resistance due to sildenafil-mediated downregulation of ABCG2 and P-gp in breast cancer cells [106]. Xanthines, such as caffeine and theophylline, are also being investigated for the same purpose and have shown promising results in vitro [107]. Unfortunately, no trials have yet investigated the potential benefit of this strategy in a clinical setting.

#### Circumventing ABCG2-mediated resistance by using agents that are poor substrates

A final and more recent proposed strategy to deal with ABC transporter-induced resistance to clinically used agents had been the development of agents that are poor substrates for efflux pumps. This strategy is having an immediate impact in the clinic. In 2010, the FDA approved the use of a novel semi-synthetic taxane derivative, cabazitaxel, in patients with castration-resistant prostate cancer who had previously failed docetaxel-based regimens [108]. Cabazitaxel was originally selected for clinical testing based in part on its poor affinity for the drug efflux pump P-glycoprotein 1 (P-gp, also known as multidrug resistance protein 1 and ABCB1) [108]. In a randomized open-label phase III trial in patients whose disease had progressed following docetaxel, cabazitaxel plus oral prednisone was superior to mitoxantrone plus oral prednisone in terms of both overall survival and progression-free survival [109]. Cabazitaxel serves as an encouraging “proof of concept” that suggests that this strategy of circumventing resistance using agents that are poor substrates for drug efflux pumps could be expanded to other ABC transporters, including ABCG2, to address the increasingly complex issue of drug failure.

Several research groups have attempted to identify anti-cancer agents that are poor substrates for ABCG2. As early as 2004, researchers in Japan were attempting to identify derivatives of camptothecin that were poor substrates of ABCG2 in the hopes of addressing irinotecan and topotecan resistance [110]. Other groups in the United States and Europe followed suit, though only a small handful of compounds have since been identified [111–113], and none have yet progressed to clinical trials. Our group recently reported on a novel semi-synthetic analogue of camptothecin, FL118 (10,11-methylenedioxy-20(S)-camptothecin), that has strong anti-cancer activity both in vitro and in vivo [114–116]. FL118 is better able to control tumor growth compared to both irinotecan and topotecan in a number of xenograft models, and has picomolar EC50 values for growth inhibition in a number of cell culture models [114, 117]. More recently, we have also reported that FL118 is a poor substrate for both ABCG2 [118] and P-gp [119]. High expression of ABCG2 and/or P-gp did not result in FL118 resistance in cell culture, and FL118 was able to inhibit tumor growth better than irinotecan in xenograft models that expressed high levels of ABCG2 [118]. Subsequently, we also observed that FL118 could eradicate xenografts that had acquired resistance to either irinotecan or topotecan [119]. This suggests that FL118 may be able to restore therapeutic efficacy in patients who had originally benefitted from irinotecan or topotecan but who later stopped responding due to acquired resistance mediated by drug efflux pumps.

Lastly, researchers who are developing the next generation of PDT agents have been particularly attracted to the notion of designing agents that are not susceptible to efflux by ABC transporters. Although a number of PDT agents are known substrates of ABCG2, including clinically used compounds, several groups had previously noted that affinity for ABCG2 was not a universally shared characteristic of common PDT drug classes [39, 41]. More recently, one group from Norway reported on amphiphilic sulfonated photosensitizers that were not effluxed out of breast cancer cells with high ABCG2 expression [120]. Specifically, they investigated sulfonated members of PDT agents in different classes, including porphines, chlorins, and phtalocyanines, and found that none of them were effluxed by ABCG2. Furthermore, another group was able to demonstrate that the potency of certain sugar-conjugated analogues of the clinically investigated HPPH were not affected by ABCG2 [42]. Taken together, these studies suggest that modifications to already-proven classes of PDT agents may produce compounds that are both efficacious and insensitive to ABCG2 activity.

## Conclusions

ABCG2 has been linked to treatment failure and decreased survival in a number of different cancers. In addition to the well-known effects of ABCG2 on cytotoxics and targeted agents, ABCG2 is also increasingly linked with failure of PDT, while the role of ABCG2 in resistance to radiation therapy remains to be further investigated. ABCG2 has also been linked to cancer cells that exhibit stem-like properties. As a result, intensive research efforts have been expended in the search for options to reverse or circumvent ABCG2-mediated resistance. In the past, most research was dedicated to identifying pharmacologic agents that would inhibit ABCG2 activity, with the hypothesis that concomitant treatment with ABCG2 inhibitors and conventional chemotherapy would increase treatment efficacy. In recent years, however, we begin to see a transition toward other strategies. Though none of these ABCG2-specific efforts have yet resulted in improvements in patient care, the broader concept of circumventing drug efflux pump-mediated resistance has led to the development and approval of cabazitaxel for prostate cancer. Encouragingly, a number of anti-cancer agents that are not substrates for ABC transporters are currently in preclinical development. FL118, for example, is a unique camptothecin analogue that is not effluxed by either ABCG2 or P-gp, and is able to overcome resistance to irinotecan and topotecan in cancer xenograft models. A number of other molecules are also currently in preclinical development, also with the goal of providing treatment options to patients who had initially responded to conventional therapy options but who had developed resistance due to increased efflux pump activity.

## Abbreviations

ABC: ATP-binding cassette
ABCG2: ATP-binding cassette, subfamily G, isoform 2 protein
ALA: 5-aminolevulinic acid
BCRP: Breast cancer resistance protein
CSC: Cancer stem cell
HPPH: 2-(1-hexyloxethyl)-2-devinyl pyropheophorbide
PDT: Photodynamic therapy
P-gp: P-glycoprotein
RNAi: RNA interference
